# Supplementary material for: Comparative Efficacy and Safety of Lorlatinib and Alectinib for ALK-Rearrangement Positive Advanced Non-Small Cell Lung Cancer in Asian and Non-Asian Patients: A Systematic Review and Network Meta-Analysis
Source: Cancers (Basel). 2021 Jul 23;13(15):3704. doi: 10.3390/cancers13153704 (PMC8345181; doi:10.3390/cancers13153704)
Supplement: Supplementary file 1 [file cancers-13-03704-s001.zip › cancers-1294387-supplementary.pdf]

Table S1. Key inclusion criteria of the studies included in this systematic review and network meta-analysis

Study name yr. [ref]	Key inclusion criteria
PROFILE1014 2014 [60]	・18 years of age or older<br>・Locally advanced, recurrent, or metastatic non-squamous ALK-p NSCLC<br>・Received no previous systemic treatment for advanced disease<br>・ECOG-PS of 0–2
PROFILE1029 2018 [61]	・Aged 18 to 70 years<br>・Locally advanced, recurrent, or metastatic non-squamous ALK-p NSCLC<br>・Received no previous systemic treatment for advanced disease<br>・ECOG-PS of 0–2
ACEND-4 2017 [63]	・18 years of age or older<br>・Locally advanced or metastatic non-squamous ALK-p NSCLC<br>・Untreated with any systemic anticancer therapy<br>・WHO-PS of 0–2
ALEX 2017 [22]	・18 years of age or older<br>・Advanced ALK-p NSCLC<br>・No previous systemic treatment for advanced NSCLC<br>・ECOG-PS of 0–2
J-ALEX 2017 [23]	・20 years of age or older<br>・Stage ⅢB, Ⅳ, or post-operative recurrent ALK-p NSCLC<br>・ALK-inhibitor-naïve Japanese patients<br>・Chemotherapy naïve or who had received one previous chemotherapy regimen<br>・ECOG-PS of 0–2
ALESIA 2019 [62]	・18 years of age or older<br>・Stage ⅢB, ⅣALK-p NSCLC<br>・Did not receive previous systemic therapy for advanced NSCLC<br>・ECOG-PS of 0–2
ALTA-1L 2018 [64]	・18 years of age or older<br>・Locally advanced or metastatic ALK-p NSCLC

	･Did not previously receive ALK-targeted therapy
CROWN 2020 [33]	･≥18 or ≥20 years of age, according to local regulations, or older<br>･Locally advanced or metastatic ALK-p NSCLC<br>･No previous systemic treatment for metastatic disease<br>･ECOG-PS of 0–2

yr, year; ref, reference number; ALK, anaplastic lymphoma kinase; ALK-p, anaplastic lymphoma kinase rearrangement positive; NSCLC, non-small cell lung cancer; ECOG-PS, Eastern Cooperative Oncology Group Performance Status; WHO-PS, World Health Organization performance status.

Table S2. Characteristics of the included studies

Study name yr. [ref]	Treatment arms	N	Age-yr. median (range)	Female No. (%)	ECOG PS No. (%)	Histologic type No. (%)	Stage of disease at entry No.(%)	CNS metastasis No. (%)	PE	Race No. (%)
PROFILE1014 2014 [60]	Pem 500 mg/m² plus [Cis 75 mg/m² or Carbo AUC = 5–6] e3w	171	54 (19–78)	108 (63)	0–1: 163 (95)<br>2: 8 (5)	Ade 161 (94)<br>Non-ade 10 (6)	LA 3 (2)<br>Meta 168 (98)	47 (27)	PFS	White 85 (50)<br>Asian 80 (47)<br>Other 6 (4)
	Criz 250 mg twice daily	172	52 (22–76)	104 (60)	0–1: 161 (94)<br>2: 10 (6)	Ade 161 (94)<br>Non-ade 11 (6)	LA 4 (2)<br>Meta 168 (98)	45 (26)		White 91 (53)<br>Asian 77 (45)<br>Other 4 (2)
		343/total								
PROFILE1029 2018 [61]	Pem 500 mg/m² plus [Cis 75 mg/m² or Carbo AUC = 5–6] e3w	103	50 (23–69)	60 (58.3)	0–1: 99 (96.1)<br>2: 4 (3.9)	Ade 101 (98.1)<br>Lar 1 (1.0)<br>Ade-squ 1 (1.0)	LA 7 (6.8)<br>Meta 96 (93.2)	32 (31.1)	PFS	Asian 103 (100)
	Criz 250 mg twice daily	104	48 (24–67)	54 (51.9)	0–1: 100 (96.2)<br>2: 4 (3.8)	Ade 100 (96.2)<br>Large 0<br>Ade-squ 4 (3.8)	LA 13 (12.5)<br>Meta 91 (87.5)	21 (20.2)		Asian 104 (100)
		207/total								
ASCEND-4 2017 [63]	Pem 500 mg/m² plus [Cis 75 mg/m² or Carbo AUC = 5–6] e3w	187	54.0 (22–80)	114 (61)	0: 70 (37)*<br>1: 105 (56)*<br>2: 11 (6)*<br>MS: 1 (1)*	Ade 183 (98)	LA 5 (3)<br>Meta 182 (97)	62 (33)	PFS	Asian 82 (44)<br>Caucasian 98 (52)<br>Other 7 (4)

	Ceri 750 mg/day<br>orally	189	55.0 (22–81)	102 (54)	0: 69 (37)*<br>1: 107 (57)*<br>2: 13 (7)*<br>MS: 0 (0)*	Ade 180 (95)	LA 9 (5)<br>Meta 180 (95)	59 (31)		Asian 76 (40)<br>Caucasian 104 (55)<br>Other 9 (5)
		376/total								
ALEX<br>2017 [22]	Alec 600mg<br>twice daily	152	58 (25–88)	84 (55)	0–1: 142 (93)<br>2: 10 (7)	Ade 137 (90)<br>Squ 5 (3)<br>Other 10 (7)	ⅢB 4 (3)<br>Ⅳ 148 (97)	64 (42)	PFS	Asian 69 (45)<br>Non-Asian 83 (55)
	Criz 250 mg<br>twice daily	151	54 (18–91)	87 (58)	0–1: 141 (93)<br>2: 10 (7)	Ade 142 (94)<br>Squ 2 (1)<br>Other 7 (5)	ⅢB 6 (4)<br>Ⅳ 145 (96)	58 (38)		Asian 69 (46)<br>Non-Asian 82 (54)
		303/total								
J-ALEX<br>2017 [23]	Alec 300mg<br>twice daily	103	61.0 (27–85)	62 (60)	0–1: 101 (98)<br>2: 2 (2)	Ade 100 (97)<br>Squ 2 (2)<br>Other 1 (1)	ⅢB 3 (3)<br>Ⅳ 76 (74)<br>POR 24 (23)	14 (14)	PFS	JP 103 (100)
	Criz 250 mg<br>twice daily	104	59.5 (25–84)	63 (61)	0–1: 102 (98)<br>2: 2 (2)	Ade 103 (99)<br>Squ 0 (0)<br>Other 1 (1)	ⅢB 3 (3)<br>Ⅳ 75 (72)<br>POR 26 (25)	29 (28)		JP 104 (100)
		207/total								
ALESIA<br>2019 [62]	Alec 600mg<br>twice daily	125	51 (43–59)**	61 (49)	0–1: 121 (97)<br>2: 4 (3)	Ade 117 (94)	ⅢB 13 (10)<br>Ⅳ 112 (90)	42 (34)	PFS	Asian 125 (100)

	Criz 250 mg<br>twice daily	62	49 (41–59)**	28 (45)	0–1: 61 (98)<br>2: 1 (2)	Ade 59 (97)	ⅢB 4 (7)<br>Ⅳ 58 (94)	20 (32)		Asian 62 (100)
		187/total								
ALTA-1L<br>2018 [64]	Brig 180 mg<br>once daily<br>(7-day run-in<br>period of 90mg<br>once daily)	137	58 (27–86)	69 (50)	0–1: 131 (96)<br>2: 6 (4)	Ade 126 (92)<br>Squ 4 (3)<br>Other 7 (4)	ⅢB 8 (6)<br>Ⅳ 129 (94)	40 (29)	PFS	Non-Asian 78 (57)<br>Asian 59 (43)
	Criz 250 mg<br>twice daily	138	60 (29–89)	81 (59)	0–1: 132 (96)<br>2: 6 (4)	Ade 137 (99)<br>Squ 0 (0)<br>Other 1 (1)	ⅢB 12 (9)<br>Ⅳ 126 (91)	41 (30)		Non-Asian 89 (64)<br>Asian 49 (36)
		275/total								
CROWN<br>2020 [33]	Criz 250 mg<br>twice daily	147	56 (45–66)**	91 (62)	0: 57 (39)<br>1: 81 (55)<br>2: 9 (6)	Ade 140 (95)<br>Ade-squ 5 (3)<br>Lar 1 (1)<br>Squ 1 (1)	ⅢA 0 (0)<br>ⅢB 8 (5)<br>Ⅳ139 (95)<br>other 0 (0)	40 (27)	PFS	White 72 (49)<br>Asian 65 (44)<br>Black 1 (1)<br>Missing 9 (6)
	Lorl 100 mg/day<br>orally	149	61 (51–69)**	84 (56)	0: 67 (45)<br>1: 79 (53)<br>2: 3 (2)	Ade 140 (94)<br>Ade-squ 6 (4)<br>Lar 0 (0)<br>Squ 3 (2)	ⅢA 1 (1)<br>ⅢB 12 (8)<br>Ⅳ135 (91)<br>other 1 (1)	38 (26)		White 72 (48)<br>Asian 65 (44)<br>Black 0 (0)<br>Missing 12 (8)
		296/total								

Note; *, WHO-PS; **, Interquartile range.

yr, year; ref, reference number; N, number of patients included in the treatment arm; No., number of patients; ECOG, Eastern Cooperative Oncology Group; PS, performance status; CNS, central nervous system; PE, primary endpoint; Pem, pemetrexed; Cis, cisplatin; Carbo, carboplatin; e3w, every 3 week; AUC, area under the curve; Ade, adenocarcinoma; Non-Ade, non-adenocarcinoma; LA, locally advanced; Meta, metastasis; PFS, progression-free survival; Criz, crizotinib; Lar, large; Ade-squ, adeno-squamous; Ceri, ceritinib; Alec, alectinib; POR, post-operative recurrence; JP, Japanese; squ, squamous; Brig, brigatinib; Lorl, lorlatinib; WHO-PS, World Health Organization performance status.

Table S3. Comparative efficacy of each pair of the six treatment arms, including chemotherapy, crizotinib, ceritinib, alectinib, brigatinib, and lorlatinib with respect to PFS, OS, and ObR.

Treatment comparisons	PFS	OS	ObR
Criz vs. Chem	0.431 (0.349–0.532)	0.819 (0.536–1.249)	4.578 (3.126–6.706)
Ceri vs. Chem	0.550 (0.418–0.726)	0.729 (0.498–1.075)	7.210 (4.591–11.40)
Alec vs. Chem	0.163 (0.116–0.227)	0.501 (0.278–0.902)	9.521 (5.377–16.82)
Brig vs. Chem	0.211 (0.134–0.333)	0.801 (0.361–1.780)	7.361 (3.916–13.85)
Lorl vs. Chem	0.121 (0.078–0.187)	0.590 (0.292–1.185)	10.49 (5.583–19.61)
Ceri vs. Criz	1.276 (0.904–1.808)	0.889 (0.504–1.586)	1.574 (0.874–2.861)
Alec vs. Criz	0.378 (0.291–0.490)	0.611 (0.407–0.918)	2.080 (1.365–3.167)
Brig vs. Criz	0.490 (0.327–0.733)	0.979 (0.498–1.923)	1.609 (0.974–2.659)
Lorl vs. Criz	0.280 (0.191–0.411)	0.721 (0.413–1.256)	2.292 (1.391–3.768)
Alec vs. Ceri	0.296 (0.191–0.456)	0.686 (0.338–1.384)	1.320 (0.635–2.726)
Brig vs. Ceri	0.384 (0.224–0.653)	1.102 (0.451–2.664)	1.022 (0.466–2.217)
Lorl vs Ceri	0.220 (0.131–0.367)	0.810 (0.363–1.792)	1.454 (0.668–3.140)
Brig vs. Alec	1.296 (0.801–2.098)	1.601 (0.726–3.527)	0.773 (0.401–1.490)
Lorl vs. Alec	0.742 (0.466–1.180)	1.180 (0.590–2.354)	1.102 (0.572–2.115)
Lorl vs. Brig	0.572 (0.326–0.997)	0.736 (0.305–1.759)	1.424 (0.699–2.886)

Comparative efficacy of each pair of treatments across six therapeutic regimens, including Lorl, Brig, Alec, Ceri, Criz, and Chem, in terms of PFS, OS, and ObR for ALP-p, ALK-inhibitor naïve advanced NSCLC. All eight studies were included for analyzing PFS and ObR, but only six studies (PROFILE1014, ASCEND-4, ALEX, ALESIA, ALTA-1L, and CROWN) were included for analyzing OS. Data are expressed as hazard ratios (HRs) and 95% credible intervals (CrIs) for PFS and OS, and expressed as odds ratios (ORs) and 95% credible intervals (CrIs) for ObR. The results of the comparison between lorlatinib and other therapeutic agents for PFS and OS are also presented visually in Figure 4 and Figure 7 of the main manuscript file, respectively.

PFS, progression-free survival; OS, overall survival; ObR, proportion of objective response; Lorl, lorlatinib; Brig, brigatinib; Alec, alectinib; Ceri, ceritinib; Criz, crizotinib; Chem, chemotherapy; ALK, anaplastic lymphoma kinase; ALK-p, anaplastic lymphoma kinase rearrangement positive; NSCLC, non-small cell lung cancer.

Table S4. SUCRA (rank) in overall participants for PFS, OR, and ObR.

Treatment regimens	PFS	OS	ObR
Chem	0.0 (6)	12.2 (6)	0.0 (6)
Criz	38.3 (4)	35.5 (5)	22.0 (5)
Ceri	21.7 (5)	52.6 (3)	56.3 (4)
Alec	79.2 (2)	87.9 (1)	78.7 (2)
Brig	63.4 (3)	40.2 (4)	57.5 (3)
Lorl	97.4 (1)	71.5 (2)	85.5 (1)

The data presented are the surface under the cumulative ranking curve (SUCRA) for efficacy in terms of PFS, OS, and ObR for the six therapeutic regimens (Lorl, Brig, Alec, Ceri, Criz, Chem) in patients with ALP-p, ALK-inhibitor naïve advanced NSCLC. All eight studies were included for analyzing PFS and ObR, but only six studies (PROFILE1014, ASCEND-4, ALEX, ALESIA, ALTA-1L, and CROWN) were included for analyzing OS. The data are listed as SUCRA values (rank) and higher SUCRA values indicate better outcomes.

PFS, progression-free survival; OS, overall survival; ObR, proportion of objective response; Lorl, lorlatinib; Brig, brigatinib; Alec, alectinib; Ceri, ceritinib; Criz, crizotinib; Chem, chemotherapy; ALK, anaplastic lymphoma kinase; ALK-p, anaplastic lymphoma rearrangement positive; NSCLC, non-small cell lung cancer.

Table S5. Comparison of efficacy of each pair of the six treatment arms, including chemotherapy, crizotinib, ceritinib, alectinib, brigatinib, and lorlatinib in terms of PFS in non-Asian and Asian patient subgroups.

Treatment comparisons	non-Asian	Asian
Criz vs. Chem	0.529 (0.365–0.769)	0.418 (0.324–0.540)
Ceri vs. Chem	0.440 (0.297–0.654)	0.659 (0.412–1.063)
Alec vs. Chem	0.259 (0.147–0.459)	0.138 (0.091–0.209)
Brig vs. Chem	0.286 (0.153–0.534)	0.171 (0.079–0.370)
Lorl vs. Chem	0.101 (0.052–0.193)	0.196 (0.107–0.363)
Ceri vs. Criz	0.829 (0.485–1.434)	1.578 (0.924–2.712)
Alec vs. Criz	0.490 (0.319–0.751)	0.331 (0.239–0.457)
Brig vs. Criz	0.540 (0.327–0.891)	0.410 (0.198–0.849)
Lorl vs. Criz	0.190 (0.112–0.324)	0.471 (0.270–0.818)
Alec vs. Ceri	0.590 (0.294–1.174)	0.209 (0.111–0.392)
Brig vs. Ceri	0.651 (0.309–1.358)	0.260 (0.104–0.643)
Lorl vs Ceri	0.229 (0.107–0.489)	0.298 (0.137–0.643)
Brig vs. Alec	1.101 (0.569–2.129)	1.239 (0.557–2.756)
Lorl vs. Alec	0.388 (0.195–0.769)	1.423 (0.748–2.708)
Lorl vs. Brig	0.352 (0.169–0.732)	1.148 (0.456–2.860)

Comparison of efficacy—in terms of PFS—of each pair of treatments across six therapeutic regimens, including Lorl, Brig, Alec, Ceri, Criz, and Chem for ALP-p ALK-inhibitor-naïve advanced NSCLC in subgroups containing Asian and non-Asian patients. Only five studies (PROFILE1014, ASCEND-4, ALEX, ALTA-1L, and CROWN) were included for analyzing the subgroups containing non-Asian patients, while all eight studies were included for analyzing the subgroups containing Asian patients. Data are expressed as hazard ratios (HRs) and 95% credible intervals (CrIs). With respect to the data presented in this table, the results of the comparison between lorlatinib and other therapeutic agents are also presented visually in Figure 5a and 5b.

PFS, progression-free survival; Lorl, lorlatinib; Brig, brigatinib; Alec, alectinib; Ceri, ceritinib; Criz, crizotinib; Chem, chemotherapy; ALK, anaplastic lymphoma kinase; ALK-p, anaplastic lymphoma kinase rearrangement positive; NSCLC, non-small cell lung cancer.

Table S6. SUCRA (rank) in non-Asian and Asian patient subgroups for PFS.

Treatment regimens	non-Asian	Asian
Chem	0.0 (6)	0.9 (6)
Criz	25.2 (5)	39.3 (4)
Ceri	38.8 (4)	20.1 (5)
Alec	71.0 (2)	91.2 (1)
Brig	65.1 (3)	78.1 (2)
Lorl	99.9 (1)	70.4 (3)

The data presented are the surface under the cumulative ranking curve (SUCRA) for efficacy regarding PFS of the six therapeutic regimens (Lorl, Brig, Alec, Ceri, Criz, Chem) in patients with ALP-p ALK-inhibitor-naïve advanced NSCLC in non-Asian and Asian patient subgroups. Only five studies (PROFILE1014, ASCEND-4, ALEX, ALTA-1L, and CROWN) were included for analyzing subgroups containing non-Asian patients, while all eight studies were included for analyzing subgroups containing Asian patients. The data are listed as SUCRA values (rank) and higher SUCRA values indicate better outcomes.

PFS, progression-free survival; Lorl, lorlatinib; Brig, brigatinib; Alec, alectinib; Ceri, ceritinib; Criz, crizotinib; Chem, chemotherapy; ALK, anaplastic lymphoma kinase; ALK-p, anaplastic lymphoma kinase rearrangement positive; NSCLC, non-small cell lung cancer.

Table S7. Comparison of efficacy of each pair of six treatment arms, including chemotherapy, crizotinib, ceritinib, alectinib, brigatinib, and lorlatinib in terms of PFS in patient subgroups with and without CNS metastasis.

Treatment comparisons	with CNS metastasis	without CNS metastasis
Criz vs. Chem	0.542 (0.367–0.799)	0.423 (0.332–0.539)
Ceri vs. Chem	0.699 (0.440–1.119)	0.480 (0.338–0.685)
Alec vs. Chem	0.200 (0.110–0.366)	0.192 (0.127–0.291)
Brig vs. Chem	0.108 (0.044–0.267)	0.304 (0.176–0.527)
Lorl vs. Chem	0.108 (0.047–0.248)	0.135 (0.081–0.226)
Ceri vs. Criz	1.289 (0.707–2.375)	1.133 (0.740–1.742)
Alec vs. Criz	0.369 (0.233–0.585)	0.455 (0.325–0.636)
Brig vs. Criz	0.200 (0.088–0.452)	0.720 (0.439–1.178)
Lorl vs. Criz	0.200 (0.097–0.414)	0.320 (0.205–0.501)
Alec vs. Ceri	0.286 (0.133–0.611)	0.401 (0.232–0.690)
Brig vs. Ceri	0.155 (0.056–0.428)	0.636 (0.329–1.219)
Lorl vs Ceri	0.155 (0.060–0.398)	0.283 (0.152–0.523)
Brig vs. Alec	0.541 (0.212–1.383)	1.582 (0.870–2.876)
Lorl vs. Alec	0.542 (0.229–1.285)	0.705 (0.402–1.234)
Lorl vs. Brig	1.003 (0.333–2.979)	0.445 (0.227–0.864)

Comparative efficacy of each pair of treatments across six therapeutic regimens, including Lorl, Brig, Alec, Ceri, Criz, and Chem in terms of PFS for ALP-p ALK-naïve advanced NSCLC in patients with and without CNS metastasis. Seven studies each (PROFILE1014, PROFILE1029, ASCEND-4, ALEX, J-ALEX, ALTA-1L, and CROWN) were included for analyzing subgroups containing patients with and without CNS metastasis. ALESIA could not be included in this analysis due to missing data on central nervous system metastases for the primary endpoint of investigator-assessed PFS. Data are expressed as hazard ratios (HRs) and 95% credible intervals (CrIs). The data presented in this table, with regard to the results of the comparison between lorlatinib and other therapeutic agents, are also presented visually in Figure 6a and 6b; CNS, central nervous system; PFS, progression-free survival; Lorl, lorlatinib; Brig, brigatinib; Alec, alectinib; Ceri, ceritinib; Criz, crizotinib; Chem, chemotherapy, HR, hazard ratio; CrI, credible interval; ALK, anaplastic lymphoma kinase; ALK-p, anaplastic lymphoma kinase positive; NSCLC, non-small cell lung cancer.

Table S8. SUCRA (rank) in the patient subgroups with and without CNS metastasis for PFS.

Treatment regimens	with CNS metastasis	without CNS metastasis
Chem	1.4 (6)	0.0 (6)
Criz	35.9 (4)	36.2 (4)
Ceri	22.7 (5)	27.4 (5)
Alec	63.6 (3)	80.9 (2)
Brig	88.0 (2)	57.9 (3)
Lorl	88.3 (1)	97.6 (1)

The data presented are the surface under the cumulative ranking curve (SUCRA) for efficacy regarding PFS of the six therapeutic regimens (Lorl, Brig, Alec, Ceri, Criz, Chem) in patients with ALP-p ALK-inhibitor–naïve advanced NSCLC in patient subgroups with and without CNS metastasis. Seven studies each (PROFILE1014, PROFILE1029, ASCEND-4, ALEX, J-ALEX, ALTA-1L, and CROWN) were for analyzing subgroups containing patients with and without CNS metastasis. ALESIA could not be included in this analysis due to missing data on central nervous system metastases for the primary endpoint of investigator-assessed PFS. The data are listed as SUCRA values (rank) and higher SUCRA values indicate better outcomes. PFS, progression-free survival; Lorl, lorlatinib; Brig, brigatinib; Alec, alectinib; Ceri, ceritinib; Criz, crizotinib; Chem, chemotherapy, HR, hazard ratio; CrI, credible interval; ALK, anaplastic lymphoma kinase; ALK, anaplastic lymphoma kinase rearrangement positive; NSCLC, non-small cell lung cancer.

Table S9. Comparative efficacy of each pair of five treatment arms, including chemotherapy, crizotinib, alectinib, brigatinib, and lorlatinib in terms of PFS in patient subgroups with PS of 0–1.

Treatment comparisons	HR (95% CrI)
Criz vs. Chem	0.433 (0.349–0.536)
Alec vs. Chem	0.157 (0.113–0.217)
Brig vs. Chem	0.215 (0.131–0.354)
Lorl vs. Chem	0.121 (0.077–0.190)
Alec vs. Criz	0.362 (0.282–0.463)
Brig vs. Criz	0.497 (0.318–0.777)
Lorl vs. Criz	0.280 (0.188–0.416)
Brig vs. Alec	1.376 (0.826–2.290)
Lorl vs. Alec	0.774 (0.486–1.233)
Lorl vs. Brig	0.562 (0.310–1.025)

Comparative efficacy of each pair of treatments across five therapeutic regimens, including Lorl, Brig, Alec, Criz, and Chem in terms of PFS for ALP-p ALK-naïve advanced NSCLC in patients with PS of 0–1. Seven studies (PROFILE1014, PROFILE1029, ALEX, J-ALEX, ALESIA, ALTA-1L, and CROWN) were included for analyzing subgroups containing patients with PS of 0–1. ASCEND-4 could not be included due to missing data required for this subgroup analysis. Data are expressed as hazard ratios (HRs) and 95% credible intervals (CrIs); PFS, progression-free survival; Lorl, lorlatinib; Brig, brigatinib; Alec, alectinib; Criz, crizotinib; Chem, chemotherapy; ALK, anaplastic lymphoma kinase; ALK-p, anaplastic lymphoma kinase positive; NSCLC, non-small cell lung cancer.

Table S10. SUCRA (rank) for PFS in the patient subgroups with PS of 0–1.

Therapeutic regimens	SUCRA (rank)
Chem	0.0 (5)
Criz	25.0 (4)
Ceri	NE
Alec	75.8 (2)
Brig	53.5 (3)
Lorl	95.7 (1)

The data presented are the surface under the cumulative ranking curve (SUCRA) for efficacy regarding PFS of the five therapeutic regimens (Lorl, Brig, Alec, Criz, Chem) in patients with ALP-p ALK-inhibitor–naïve advanced NSCLC in patient subgroups with a PS of 0–1. Seven studies (PROFILE1014, PROFILE1029, ALEX, J-ALEX, ALESIA, ALTA-1L, and CROWN) were included for analyzing subgroups containing patients with PS of 0-1. ASCEND-4 could not be included due to missing data required for this subgroup analysis. The data are listed as SUCRA values (rank) and higher SUCRA values indicate better outcomes; PFS, progression-free survival; Lorl, lorlatinib; Brig, brigatinib; Alec, alectinib; Ceri, ceritinib; Criz, crizotinib; Chem, chemotherapy, HR, hazard ratio; CrI, credible interval; ALK, anaplastic lymphoma kinase; ALK, anaplastic lymphoma kinase rearrangement positive; NSCLC, non-small cell lung cancer; NE, not evaluable.

Table S11. Comparative safety of each pair of four treatment arms, including crizotinib, alectinib, brigatinib, and lorlatinib in terms of AG-AEs, G3-AEs, AG-SAEs, and G3-SAEs.

	AEs		SAEs	
Treatment comparisons	AG-AEs	G3-AEs	AG-SAEs	G3-SAEs
Alec vs. Criz	0.992 (0.972–1.013)	0.678 (0.566–0.811)	0.773 (0.592–1.009)	0.970 (0.680–1.381)
Brig vs. Criz	0.970 (0.941–1.001)	1.100 (0.899–1.350)	NE	NE
Lorl vs. Criz	1.010 (0.985–1.035)	1.300 (1.085–1.554)	1.249 (0.881–1.768)	1.219 (0.816–1.818)
Brig vs. Alec	0.978 (0.942–1.015)	1.623 (1.239–2.132)	NE	NE
Lorl vs. Alec	1.018 (0.985–1.051)	1.918 (1.486–2.475)	1.614 (1.042–2.503)	1.255 (0.737–2.146)
Lorl vs. Brig	1.041 (1.001–1.083)	1.181 (0.900–1.546)	NE	NE

Comparative safety of each pair of treatments across four therapeutic regimens, including Lorl, Brig, Alec, and Criz in terms of AG-AEs, G3-AEs, AG-SAEs, and AG-SAEs for ALP-p ALK-naïve advanced NSCLC. For the analysis of AG-AEs and G3-AEs, only five studies (ALEX, J-ALEX, ALESIA, ALTA-1L, and CROWN) were available for inclusion; for the analysis of AG-SAEs, four studies (ALEX, J-ALEX, ALESIA, and CROWN) were available, and for G3-SAEs, only two studies (ALEX and CROWN) were available for inclusion. Data are expressed as risk ratios (RRs) and 95% credible intervals (CrIs). The data presented in this table, regarding the result of the comparison between lorlatinib and other therapeutic agents for G3-AEs, which was the primary safety endpoint, are also presented visually in Figure 8; AEs, any adverse events; SAEs, serious adverse events; AG-AEs, any grade of any adverse events; G3-AEs, Grade 3 or higher of adverse events; AG-SAEs, any grade of serious adverse events; G3-SAEs, Grade 3 or higher of serious adverse events; Lorl, lorlatinib; Brig, brigatinib; Alec, alectinib; Criz, crizotinib; ALK, anaplastic lymphoma kinase; ALK-p, anaplastic lymphoma kinase rearrangement positive; NSCLC, non-small cell lung cancer; NE, not evaluable.

Table S12. SUCRA (rank) in overall participants for AG-AEs, G3-AEs, AG-SAEs, and G3-SAEs

	AEs		SAEs	
Treatment regimens	AG-AEs	G3-AEs	AG-SAEs	G3-SAEs
Criz	34.9 (3)	60.7(2)	46.2 (2)	63.4 (2)
Alec	58.0 (2)	100.0 (1)	97.8 (1)	68.4 (1)
Brig	94.4 (1)	35.4 (3)	NE	NE
Lorl	12.7 (4)	3.9 (4)	6.0 (3)	18.3 (3)

The data presented are the surface under the cumulative ranking curve (SUCRA) for the safety in terms of AG-AEs, G3-AEs, AG-SAEs, and G3-SAEs for the four therapeutic regimens (Lorl, Brig, Alec, and Criz) in patients with ALP-p ALK-naïve advanced NSCLC. For the analysis of AG-AEs and G3-AEs, only five studies (ALEX, J-ALEX, ALESIA, ALTA-1L, and CROWN) were available for inclusion; for the analysis of AG-SAEs, four studies (ALEX, J-ALEX, ALESIA, and CROWN) were available, and for G3-AEs, only two studies (ALEX and CROWN) were available for inclusion. The data are listed as SUCRA values (rank). Higher SUCRA values indicate better outcomes; AEs, any adverse events; SAEs, serious adverse events; AG-AEs, any grade of any adverse events; G3-AEs, Grade 3 or higher of adverse events; AG-SAEs, any grade of serious adverse events; G3-SAEs, Grade 3 or higher of serious adverse events; Lorl, lorlatinib; Brig, brigatinib; Alec, alectinib; Criz, crizotinib; ALK, anaplastic lymphoma kinase; ALK-p, anaplastic lymphoma kinase rearrangement positive; NSCLC, non-small cell lung cancer; NE, not evaluable.

Table S13. Comparative safety of each pair of six treatment arms, including chemotherapy, crizotinib, ceritinib, alectinib, brigatinib, and lorlatinib in terms of AG-nausea, G3-nausea, AG-diarrhea, and G3-diarrhea.

	Nausea		Diarrhea	
Treatment comparisons	AG-nausea	G3-nausea	AG-diarrhea	G3-diarrhea
Criz vs. Chem	0.979 (0.841–1.141)	0.657 (0.110–3.880)	5.182 (3.669–7.302)	3.922 (0.441–34.510)
Ceri vs. Chem	1.239 (1.052–1.463)	0.508 (0.177–1.484)	7.789 (5.093–12.00)	4.608 (1.037–20.960)
Alec vs. Chem	0.214 (0.150–0.304)	0.142 (0.014–1.407)	1.138 (0.711–1.813)	0.648 (0.032–12.930)
Brig vs. Chem	0.460 (0.322–0.657)	0.328 (0.028–3.838)	4.665 (3.085–7.017)	2.625 (0.153–43.650)
Lorl vs. Chem	0.274 (0.178–0.424)	0.211 (0.011–3.840)	2.127 (1.304–3.456)	7.531 (0.291–189.60)
Ceri vs. Criz	1.265 (1.013–1.584)	0.773 (0.098–6.288)	1.503 (0.871–2.614)	1.172 (0.085–16.970)
Alec vs. Criz	0.218 (0.159–0.300)	0.216 (0.051–0.920)	0.220 (0.160–0.301)	0.165 (0.021–1.297)
Brig vs. Criz	0.470 (0.340–0.650)	0.499 (0.091–2.730)	0.900 (0.718–1.127)	0.668 (0.112–4.006)
Lorl vs. Criz	0.280 (0.186–0.421)	0.322 (0.032–3.197)	0.410 (0.290–0.580)	1.918 (0.173–21.020)
Alec vs. Ceri	0.172 (0.117–0.254)	0.278 (0.022–3.495)	0.146 (0.077–0.275)	0.141 (0.005–4.004)
Brig vs. Ceri	0.372 (0.250–0.550)	0.645 (0.043–9.310)	0.599 (0.329–1.080)	0.569 (0.023–13.570)
Lorl vs Ceri	0.221 (0.139–0.352)	0.415 (0.018–9.110)	0.273 (0.142–0.522)	1.635 (0.045–57.010)
Brig vs. Alec	2.151 (1.368–3.387)	2.304 (0.246–21.550)	4.098 (2.780–6.046)	4.042 (0.263–62.420)
Lorl vs. Alec	1.284 (0.764–2.153)	1.487 (0.097–22.690)	1.869 (1.167–2.988)	11.620 (0.482–275.70)
Lorl vs. Brig	0.597 (0.353–1.002)	0.645 (0.036–11.140)	0.456 (0.301–0.688)	2.874 (0.142–56.560)

Comparative safety of treatments of each pair across six therapeutic regimens, including Lorl, Brig, Alec, Ceri, Criz, and Chem in terms of AG-nausea, G3-nausea, AG-diarrhea, and G3-diarrhea for ALP-p ALK-naïve advanced NSCLC. All eight studies were included in the analysis of AG-nausea and AG-diarrhea, but only seven studies (PROFILE1014, ASCEND-4, ALEX, J-ALEX, ALESIA, ALTA-1L, and CROWN) were available for the analysis of G3-nausea. For the analysis of G3-diarrhea, only six studies (PROFILE1014, ASCEND-4, ALEX, J-ALEX, ALTA-1L, and CROWN) could be included. Data are expressed as risk ratios (RRs) and 95% credible

intervals (CrIs); AG-nausea, any grade of nausea; G3-AEs, Grade 3 or higher of nausea; AG-diarrhea, any grade of diarrhea; G3-diarrhea, Grade 3 or higher of diarrhea; Lorl, lorlatinib; Brig, brigatinib; Alec, alectinib; Ceri, ceritinib; Criz, crizotinib; Chem, chemotherapy; ALK, anaplastic lymphoma kinase; ALK-p, anaplastic lymphoma kinase rearrangement positive; NSCLC, non-small cell lung cancer.

Table S14. SUCRA (rank) in overall participants for AG-nausea, G3-nausea, AG-diarrhea, and G3-diarrhea.

	Nausea		Diarrhea	
Treatment regimens	AG-nausea	G3-nausea	AG-diarrhea	G3-diarrhea
Chem	27.8 (5)	16.2 (6)	94.1 (1)	77.9 (2)
Criz	31.7 (4)	29.6 (5)	22.1 (5)	34.6 (4)
Ceri	0.5 (6)	46.2 (4)	2.3 (6)	31.4 (5)
Alec	96.6 (1)	83.1 (1)	85.8 (2)	84.4 (1)
Brig	60.5 (3)	56.8 (3)	35.5 (4)	49.4 (3)
Lorl	82.9 (2)	68.0 (2)	60.1 (3)	22.2 (6)

The data presented are the surface under the cumulative ranking curve (SUCRA) for the safety in terms of AG-nausea, G3-nausea, AG-diarrhea, and G3-diarrhea for the six therapeutic regimens (Lorl, Brig, Alec, Ceri, Criz, Chem) in patients with ALP-p ALK-naïve advanced NSCLC. All eight studies were included in the analysis of AG-nausea and AG-diarrhea, but only seven studies (PROFILE1014, ASCEND-4, ALEX, J-ALEX, ALESIA, ALTA-1L, and CROWN) were available for the analysis of G3-nausea. For the analysis of G3-diarrhea, only six studies (PROFILE1014, ASCEND-4, ALEX, J-ALEX, ALTA-1L, and CROWN) could be included. The data are listed as SUCRA values (rank). Higher SUCRA values indicate better outcomes; AG-nausea, any grade of nausea; G3-nausea, Grade 3 or higher of nausea; AG-diarrhea, any grade of diarrhea; G3-diarrhea, Grade 3 or higher of diarrhea; Lorl, lorlatinib; Brig, brigatinib; Alec, alectinib; Ceri, ceritinib; Criz, crizotinib; Chem, chemotherapy; ALK, anaplastic lymphoma kinase; ALK-p, anaplastic lymphoma kinase rearrangement positive; NSCLC, non-small cell lung cancer.

Table S15. Comparative safety of each pair of four treatment arms, including crizotinib, alectinib, brigatinib, and lorlatinib in terms of AG-AST, G3-AST, AG-ALT, and G3-ALT.

Treatment comparisons	Increased AST levels		Increased ALT levels	
	AG-AST	G3-AST	AG-ALT	G3-ALT
Alec vs. Criz	0.468 (0.320–0.684)	0.448 (0.207–0.964)	0.598 (0.472–0.756)	0.261 (0.131–0.519)
Brig vs. Criz	0.920 (0.603–1.411)	0.251 (0.052–1.221)	0.600 (0.393–0.920)	0.150 (0.037–0.622)
Lorl vs. Criz	0.510 (0.315–0.819)	0.569 (0.137–2.315)	0.520 (0.342–0.786)	0.639 (0.181–2.220)
Brig vs. Alec	1.966 (1.115–3.483)	0.560 (0.098–3.245)	1.004 (0.620–1.633)	0.576 (0.121–2.785)
Lorl vs. Alec	1.089 (0.591–2.006)	1.269 (0.254–6.300)	0.869 (0.538–1.399)	2.447 (0.584–10.18)
Lorl vs. Brig	0.553 (0.291–1.046)	2.268 (0.271–18.61)	0.866 (0.476–1.563)	4.248 (0.639–27.77)

Comparative safety of each pair of treatments across four therapeutic regimens, including Lorl, Brig, Alec, and Criz in terms of AG-AST, G3-AST, AG-ALT, and G3-ALT for ALP-p ALK-naïve advanced NSCLC. Only four studies (ALEX, J-ALEX, ALTA-1L, and CROWN) could be included in the analysis of AG-AST and G3-AST, and only five studies (ALEX, J-ALEX, ALESIA, ALTA-1L, and CROWN) could be included in the analysis of AG-ALT and G3-ALT. Data are expressed as risk ratios (RRs) and 95% credible intervals (CrIs); AST, aspartate aminotransferase; ALT, alanine aminotransferase; AG-AST, any grade of increased aspartate aminotransferase levels; G3-AST, Grade 3 or higher of increased aspartate aminotransferase levels; AG-ALT, any grade of increased alanine aminotransferase levels; G3-ALT, Grade 3 or higher of increased alanine aminotransferase levels; Lorl, lorlatinib; Brig, brigatinib; Alec, alectinib; Criz, crizotinib; ALK, anaplastic lymphoma kinase; ALK-p, anaplastic lymphoma kinase rearrangement positive; NSCLC, non-small cell lung cancer.

Table S16. SUCRA (rank) in overall participants for AG-AST, G3-AST, AG-ALT, and G3-ALT.

	Increased AST levels		Increased ALT levels	
Treatment regimens	AG-AST	G3-AST	AG-ALT	G3-ALT
Criz	11.8 (4)	9.3 (4)	0.4 (4)	8.2 (4)
Alec	86.6 (1)	61.8 (2)	59.6 (3)	71.2 (2)
Brig	23.1 (3)	82.5 (1)	60.0 (2)	89.5 (1)
Lorl	78.5 (2)	46.4 (3)	80.1 (1)	31.2 (3)

The data presented are the surface under the cumulative ranking curve (SUCRA) for the safety in terms of AG-AST, G3-AST, AG-ALT, and G3-ALT for the four therapeutic regimens (Lorl, Brig, Alec, and Criz) in patients with ALP-p ALK-naïve advanced NSCLC. Only four studies (ALEX, J-ALEX, ALTA-1L, and CROWN) could be included in the analysis of AG-AST and G3-AST, and only five studies (ALEX, J-ALEX, ALESIA, ALTA-1L, and CROWN) could be included in the analysis of AG-ALT and G3-ALT. The data are listed as SUCRA values (rank). Higher SUCRA values indicate better outcomes; AST, aspartate aminotransferase; ALT, alanine aminotransferase; AG-AST any grade of increased aspartate aminotransferase levels; G3-AST, Grade 3 or higher of increased aspartate aminotransferase levels; AG-ALT, any grade of increased alanine aminotransferase levels; G3-ALT, Grade 3 or higher of increased alanine aminotransferase levels; Lorl, lorlatinib; Brig, brigatinib; Alec, alectinib; Criz, crizotinib; ALK, anaplastic lymphoma kinase; ALK-p, anaplastic lymphoma kinase rearrangement positive; NSCLC, non-small cell lung cancer.

Table S17. Comparative safety with respect to pneumonitis incidence between each pair of three treatment arms, including crizotinib, alectinib, and lorlatinib.

Treatment regimens	Pneumonitis	
	AG-pneumonitis	G3-pneumonitis
Alec vs. Criz	0.499 (0.091–2.714)	0.140 (0.008–2.296)
Lorl vs. Criz	0.946 (0.135–6.569)	0.318 (0.011–8.930)
Lorl vs. Alec	1.881 (0.145–25.02)	2.250 (0.029–177.2)

Comparative safety of each pair of treatments across three therapeutic regimens, including Lorl, Alec, and Criz in terms of AG-pneumonitis and G3-pneumonitis for ALP-p ALK-naïve advanced NSCLC. Only two studies (ALEX and CROWN) were available for inclusion in the analysis of AG-pneumonia and G3-pneumonia. Data are expressed as risk ratios (RRs) and 95% credible intervals (CrIs). For G3-pneumonitis, the range of 95% CrI was extremely wide in the Lorl vs. Alec results. This was thought to be related to the fact that the frequency of G3-pneumonitis was zero in the Lorl and Alec groups, respectively, in the ALEX and CROWN studies. Although convergence was confirmed by both the *BCR* diagnostic method and visual diagnosis, we consider this result of comparison for G3-pneumonitis of Lorl and Alec only to be informative. AG-pneumonitis, any grade of pneumonitis; G3-pneumonitis, Grade 3 or higher of pneumonitis; Lorl, lorlatinib; Alec, alectinib; Criz, crizotinib; ALK, anaplastic lymphoma kinase; ALK-p, anaplastic lymphoma kinase rearrangement positive; NSCLC, non-small cell lung cancer.

Table S18. SUCRA (rank) in overall participants for pneumonitis.

	Pneumonitis	
Treatment regimens	AG-pneumonitis	G3-pneumonitis
Criz	34.4(3)	16.7 (3)
Alec	73.7 (1)	77.9 (1)
Lorl	41.9 (2)	55.5 (2)

The data presented are the surface under the cumulative ranking curve (SUCRA) for the safety of the three therapeutic regimens (Lorl, Alec, and Criz) in terms of AG-pneumonitis and G3-pneumonitis in patients with ALP-p ALK-naïve advanced NSCLC. Only two studies (ALEX and CROWN) were available for inclusion in the analysis of AG-pneumonia and G3-pneumonia. The data are listed as SUCRA values (rank). Higher SUCRA values indicate better outcomes; AG-pneumonitis, any grade of pneumonitis; G3-AST, Grade 3 or higher of pneumonitis; Lorl, lorlatinib; Alec, alectinib; Criz, crizotinib; ALK, anaplastic lymphoma kinase; ALK-p, anaplastic lymphoma kinase rearrangement positive; NSCLC, non-small cell lung cancer.

Table S19. Sensitivity analysis performed by including only patient groups without prior systemic anticancer therapy

Treatment comparisons	HR (95% CrI)
Criz vs. Chem	0.431 (0.349–0.532)
Ceri vs. Chem	0.550 (0.418–0.726)
Alec vs. Chem	0.158 (0.114–0.220)
Brig vs. Chem	0.237 (0.141–0.398)
Lorl vs. Chem	0.121 (0.078–0.187)
Ceri vs. Criz	1.276 (0.904–1.808)
Alec vs. Criz	0.367 (0.285–0.473)
Brig vs. Criz	0.550 (0.342–0.884)
Lorl vs. Criz	0.280 (0.191–0.411)
Alec vs. Ceri	0.288 (0.187–0.441)
Brig vs. Ceri	0.431 (0.238–0.776)
Lorl vs Ceri	0.220 (0.131–0.367)
Brig vs. Alec	1.496 (0.873–2.565)
Lorl vs. Alec	0.763 (0.481–1.209)
Lorl vs. Brig	0.510 (0.275–0.936)

A sensitivity analysis was performed by excluding the group of patients with prior treatment with systemic anticancer chemotherapy included in two trials (J-ALEX and ALTA-1L), and only including patients with no prior treatment with systemic anticancer therapy. Comparative efficacy for PFS of each pair of treatments across the six therapeutic regimens, including Lorl, Brig, Alec, Ceri, Criz, and Chem for ALP-p ALK-naïve advanced NSCLC were shown. Data are expressed as hazard ratios (HRs) and 95% credible intervals (CrIs). PFS, progression-free survival; Lorl, lorlatinib; Brig, brigatinib; Alec, alectinib; Ceri, ceritinib; Criz, crizotinib; Chem, chemotherapy; HR, hazard ratio; CrI, credible interval; ALK, anaplastic lymphoma kinase; ALK, anaplastic lymphoma kinase; NSCLC, non-small cell lung cancer.

Table S20. Sensitivity analysis for ranking PFS assessment performed by including only patient groups without prior systemic anticancer therapy

Therapeutic regimens	SUCRA (rank)
Chem	0.0 (6)
Criz	38.5 (4)
Ceri	21.7 (5)
Alec	81.1 (2)
Brig	61.5 (3)
Lorl	97.2 (1)

A sensitivity analysis was performed by excluding the group of patients with prior treatment with systemic anticancer chemotherapy included in two trials (J-ALEX and ALTA-1L), and only including patients without any prior treatment with systemic anticancer therapy. The data presented are the surface under the cumulative ranking curve (SUCRA) for the efficacy of the six therapeutic regimens (Lorl, Brig, Alec, Ceri, Criz, Chem) in terms of PFS in patients with ALP-p ALK-naïve advanced NSCLC. The data are listed as SUCRA values (rank) and higher SUCRA values indicate better outcomes.

PFS, progression-free survival; Lorl, lorlatinib; Brig, brigatinib; Alec, alectinib; Ceri, ceritinib; Criz, crizotinib; Chem, chemotherapy; HR, hazard ratio; CrI, credible interval; ALK, anaplastic lymphoma kinase; ALK, anaplastic lymphoma rearrangement positive; NSCLC, non-small cell lung cancer.

Figure S1.

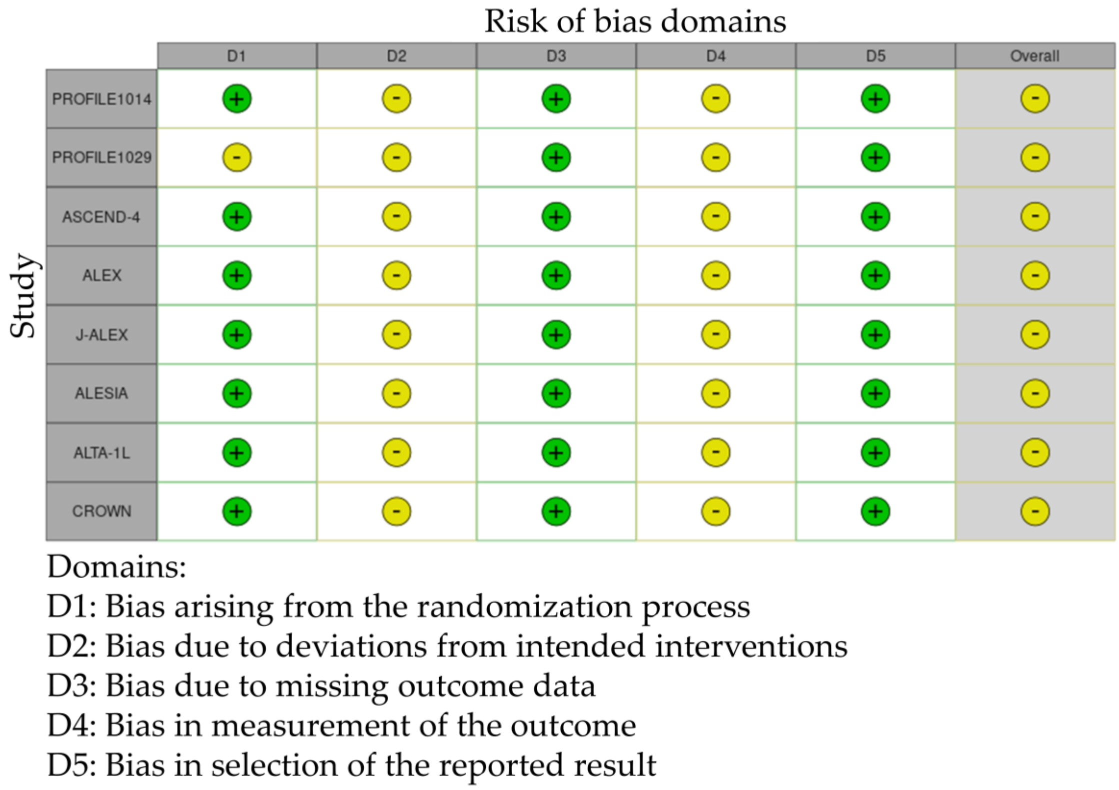

Figure S1. Risk of bias summary. The author's assessment of each risk of bias item for each incorporated study is expressed. The symbols "+", "-", and "×" indicate a low risk of bias, some concerns, and a high risk of bias, respectively. The quality of the included studies was considered generally good, as no study was assessed as having a high risk of bias. However, all eight studies included in the current systematic review and NMA were judged as having some concerns in the overall analysis. This was because these eight studies were open-label studies and were judged to have some concerns in the domain of bias due to deviation from intended intervention and bias in measurement of the outcome. Additionally, PROFILE1029 [61] was judged as having some concerns in the domain of bias arising from the randomization process owing to inadequate descriptions of the details of randomization.

Figure S2.

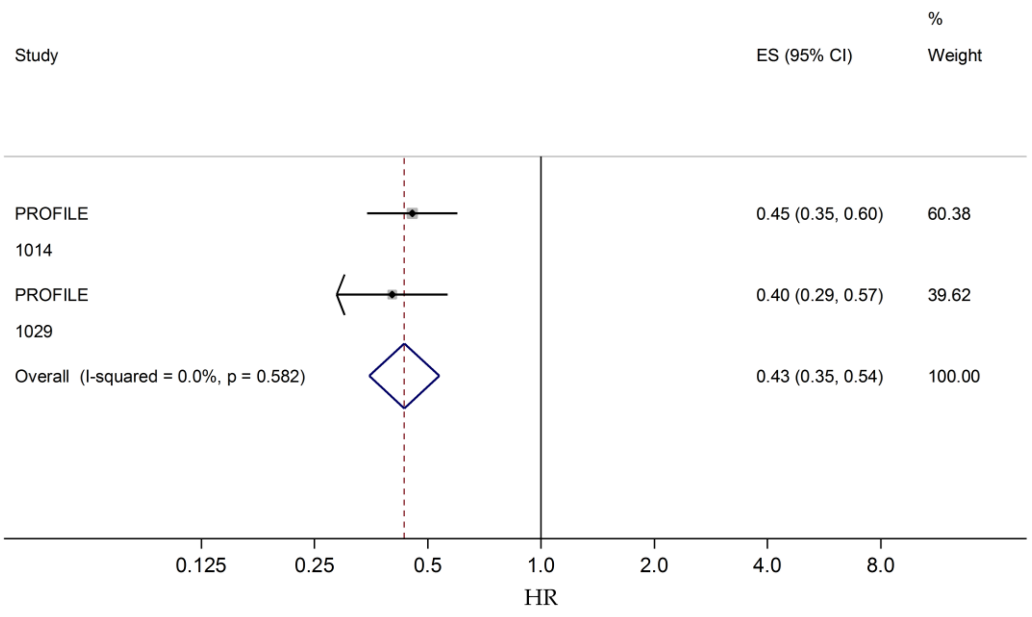

Figure S2. Forest plot for two trials comparing crizotinib and chemotherapy. A meta-analysis of two trials (PROFILE1014 [60] and PROFILE1029 [61]) comparing Criz and Chem for PFS was performed based on random effect model, with an assessment of heterogeneity being the main objective. Heterogeneity (I^2) was expressed as I-squared (%). Overall effect size (ES) for PFS was expressed as HR and 95% CI. The data has been obtained from previous studies [60, 61].

Criz, crizotinib; Chem, chemotherapy; HR, hazard ratio; CI, confidence interval; PFS, progression-free survival.

Figure S3.

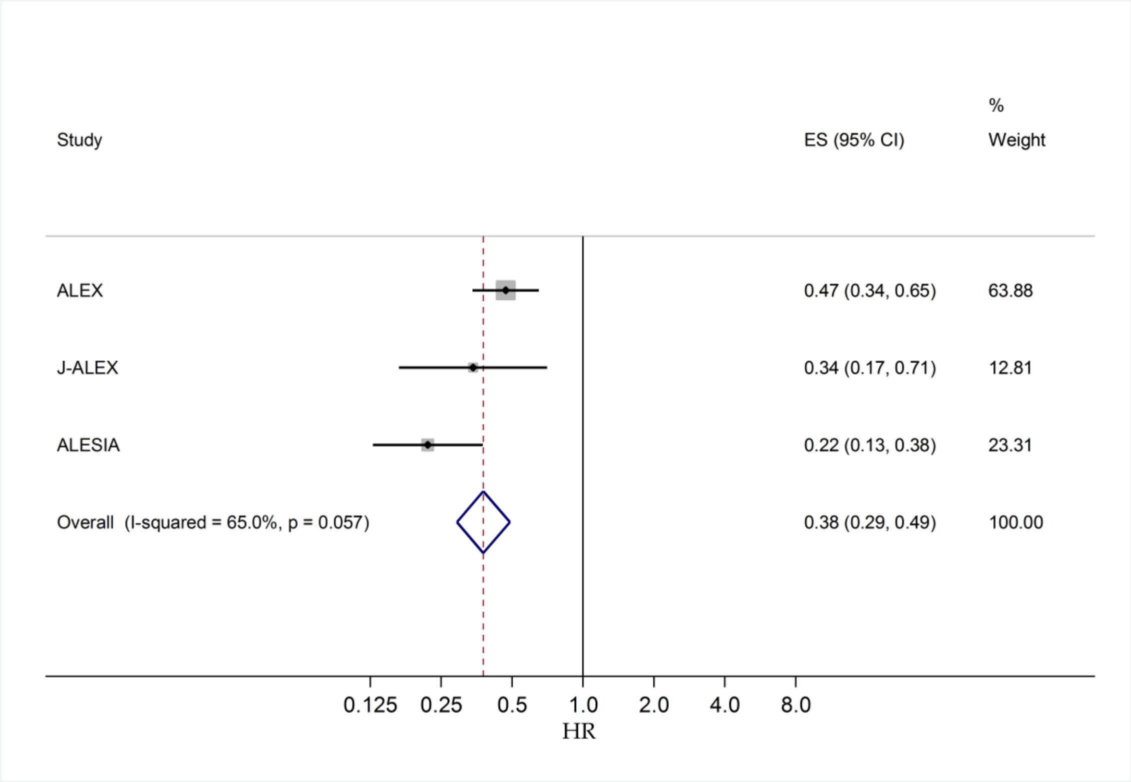

Figure S3. Forest plot of three trials comparing alectinib and crizotinib. A meta-analysis of three trials (ALEX [22], J-ALEX [23], and ALESIA [62]) comparing Alec and Criz for PFS was performed based on a random-effect model, with an assessment of heterogeneity being the main objective. Heterogeneity (I^2) was expressed as I-squared (%). Overall effect size (ES) for PFS was expressed as HR and 95% CI. The data has been obtained from previous studies [22, 23, 62].

Alec, alectinib; Criz, crizotinib; HR, hazard ratio; CI, confidence interval; PFS, progression-free survival.
